# Efficacy and safety evaluation of mixed nutrition for postoperative esophageal cancer patients in China: a meta-analysis

**DOI:** 10.3389/fonc.2024.1417765

**Published:** 2024-08-08

**Authors:** Shui Liu, Lin Qiao, Yang Liu, Hangmei Liu, Yiwen Li, Jingbo Sun, Wei Chen, Rongguo Shang, Lili Zhang, Xiaochuan Liu

**Affiliations:** ^1^ Department of Pharmacy, Emergency General Hospital, Beijing, China; ^2^ Department of Gastroenterology, Emergency General Hospital, Beijing, China

**Keywords:** mixed nutrition, parenteral nutrition, postoperative esophageal cancer, meta-analysis, efficacy and safety

## Abstract

**Objective:**

The purpose of this study was to evaluate the clinical effect of mixed nutrition and parenteral nutrition support on postoperative patients with esophageal cancer.

**Method:**

By searching PubMed, Web of Science, Cochrane, CNKI, Wanfang and other databases, all the literatures until March 2024 about the comparison of randomized controlled Trial (RCT) of mixed nutrition and parenteral nutrition support in postoperative patients with esophageal cancer were screened. The inclusion criteria were that the patients were from randomized controlled trials or clinical trials in China, and the patients were all diagnosed with esophageal cancer by pathological biopsy. The exclusion criteria were the literature other than the above, including repeated published literature, non-Chinese and English literature, incomplete or missing analysis data, etc. After two researchers independently screened the literature, extracted the data and evaluated the risk of bias according to the criteria, Meta-analysis was carried out with RevMan 5.4 software.

**Results:**

A total of 11 studies were included, including 1216 patients. Meta-analysis showed that, compared with parenteral nutrition, mixed nutrition can improve the levels of transferrin, serum albumin, prealbumin and lymphocyte counts in patients with esophageal cancer after surgery, shorten the time of anal recovery of exhaust, defecation and hospital stay after surgery, and reduce the incidence of pulmonary infection, abdominal distension, incision infection and anastomotic fistula, with statistical significance between the two groups (*P* < 0.05). The heterogeneity of individual results in this study is relatively high, the analysis comes from clinical heterogeneity, and the publication bias is analyzed through Funnel plot. Taking the incidence of lung infection as an example, the results are evenly distributed on both sides of the Funnel plot, and the publication bias has little impact on the results of the study.

**Conclusion:**

Compared with parenteral nutrition, mixed nutrition can improve the prognosis of postoperative patients with esophageal cancer and reduce the incidence of related adverse events.

## Introduction

Esophageal cancer is one of the most common malignant tumors worldwide. According to the global cancer data in 2020, there are 600,000 new cases of esophageal cancer worldwide, ranking 8th among all tumors; There were 544,000 deaths, ranking sixth among all tumors; More than half of the new cases and deaths of esophageal cancer come from China (1). In China, the number of new cases of esophageal cancer ranks sixth among all tumors, and the number of deaths ranks fourth among all tumors (2). Worldwide esophageal cancer incidence is projected to increase by 63.5% and deaths by 68% by 2040 (3).

The disease tends to occur in the esophageal epithelial tissue, and the main clinical symptoms are progressive dysphagia or conscious eating obstruction, retrosternal pain and other discomforts. Most patients are already in the middle and late stages when they are diagnosed (4). At present, the treatment methods for esophageal cancer include surgery, radiotherapy, chemotherapy, biological therapy and traditional Chinese medicine treatment, etc. According to the general condition of the patient, clinical stage, lesion location, etc., surgery is still the preferred method (5). Patients with esophageal cancer have a high incidence of malnutrition due to the special pathological changes and physiological functions of the esophagus. Literature studies (6) have reported that 60% to 85% of esophageal cancer patients have different degrees of malnutrition.

Perioperative malnutrition in patients with esophageal cancer can bring many negative effects, such as the suppression of immune function, acute inflammatory damage, etc., thereby increasing the incidence of postoperative complications and mortality. Therefore, the patients with esophageal cancer perioperative reasonable and effective nutritional support has a very positive significance (7), and early postoperative use of nutritional support has also become a research focus of scholars. At present, there have been many studies on the clinical efficacy of MN (Multinutrition) or PN (Parenteral Nutrition) in patients with esophageal cancer after operation, but the conclusions are not the same. This study used the collected research data to compare the efficacy and safety evaluation of postoperative MN and PN in Chinese esophageal cancer patients by means of Meta-analysis, aiming to fill the gaps in existing studies and provide a clearer theoretical basis for research.

## Materials and methods

The English database used “Enteral Nutrition”, “Parenteral Nutrition”, “Multi-nutrition”, “EsophagusCancer”, “randomized controlled trial”, “RCT”, etc. as search terms, and searched PubMed, EMbase, The CochraneLibrary; The Chinese database uses “enteral nutrition”, “parenteral nutrition”, “mixed nutrition”, “esophageal cancer”, “randomized controlled study”, etc. as the search words, and searches China National Knowledge Network (CNKI), Chinese scientific and technological journal database (VIP), Wanfang database, etc. After reading the abstract and the full text, the literature that did not meet the inclusion criteria was excluded, and the relevant references were consulted to check for omissions and fill in gaps. The search time limit is until March 2024.

### Inclusion criteria

The type of study must be a randomized controlled trial or clinical trial, and the patients are from China; The study subjects need to meet the pathological biopsy diagnosis of esophageal cancer, and concurrent surgical treatment, but the surgical method is not limited. In terms of intervention measures, the experimental group received enteral combined parenteral nutrition support (MN), Namely, parenteral nutrition was the same as that of the control group. On the first day after operation, normal saline was dripped into the enteral nutrition tube, and the dripping speed was adjusted according to the actual situation of the patient. On the second day after operation, enteral nutrition suspension and the control group received parenteral nutrition (PN) support were inputted through the catheter. Nutrient solution, including electrolytes, carbohydrates, amino acids and vitamins necessary for the patient’s body, was inputted from the first day after operation. The outcome indexes were ① serum transferrin (TRF); Albumin (ALB); ③ prealbumin (PA); ④ Lymphocyte count (LYM); Immunoglobulins (IgA, IgG, IgM); ⑥ anal exhaust recovery time; ⑦ Defecation time; ⑧ Length of hospital stay; ⑨ Adverse events (pulmonary infection, abdominal distension, incision infection, anastomotic fistula). The results of ①, ②, ③, ④ and ⑤ were the 7th day after operation.

### Exclusion criteria

Duplicate publications, non-Chinese and English literature, incomplete or missing analytical data, unable to be obtained by contacting the original author, and data unable to be extracted should be excluded.

### Data extraction and quality assessment

Two evaluators independently screen literature, extract data and cross-check. In case of differences, consult a third party to help judge, and contact the author as much as possible to supplement the lack of data. When selecting documents, first read the title and abstract, and then read the full text after excluding obviously irrelevant documents to determine whether to include them in the end. The data extraction contents mainly include: ① the first author/published year; ② Basic characteristics of the subject; ③ Sample size; ④ Intervention plan; ⑤ Outcome index. For the original study with multiple subgroups, the data of the experimental group and the control group related to this study were extracted.

The bias risk included in the study was evaluated by two evaluators according to the bias risk quality evaluation tool for RCT in Cochrane Manual (8). The evaluation criteria of the Cochrane Bias Risk Assessment Tool are divided into 7 items: ① Random sequence generation (selection bias); ② Allocation concealment (selection bias); ③ Blinding of participants and personnel (performance bias) ④ Blinding of outcome assessment (detection bias); ⑤ Incomplete outcome data (attrition bias); ⑥ Selective reporting (reporting bias); ⑦ Other bias, For each item, low bias, uncertain risk of bias and high bias were used to judge and divide the quality of the study.

### Statistical analysis

RevMan 5.4 software was used for Meta-analysis. The count data were analyzed by relative risk (RR), and the continuous data were analyzed by mean difference (MD) or standardized mean difference (SMD). The combined effect and its 95% confidence interval (CI) were calculated at the same time. Heterogeneity included in the study results was analyzed by χ^2^ test (the test level was α = 0.1), and the size of heterogeneity was quantitatively judged by combining I^2^. If there is no statistical heterogeneity among the research results, the fixed effect model is used for Meta-analysis; If there is statistical heterogeneity among the results, the source of heterogeneity is further analyzed. After excluding the influence of obvious clinical heterogeneity, the random effect model is used for Meta-analysis. Significant clinical heterogeneity was treated by subgroup analysis or sensitivity analysis, or only descriptive analysis. According to the recommendation of Cochrane Systematic Review Production Manual, when the number of included literatures is ≥ 10, the publication bias test is carried out by funnel diagram.

## Results

### Basic characteristics and quality evaluation of literature

**Figure 1:**
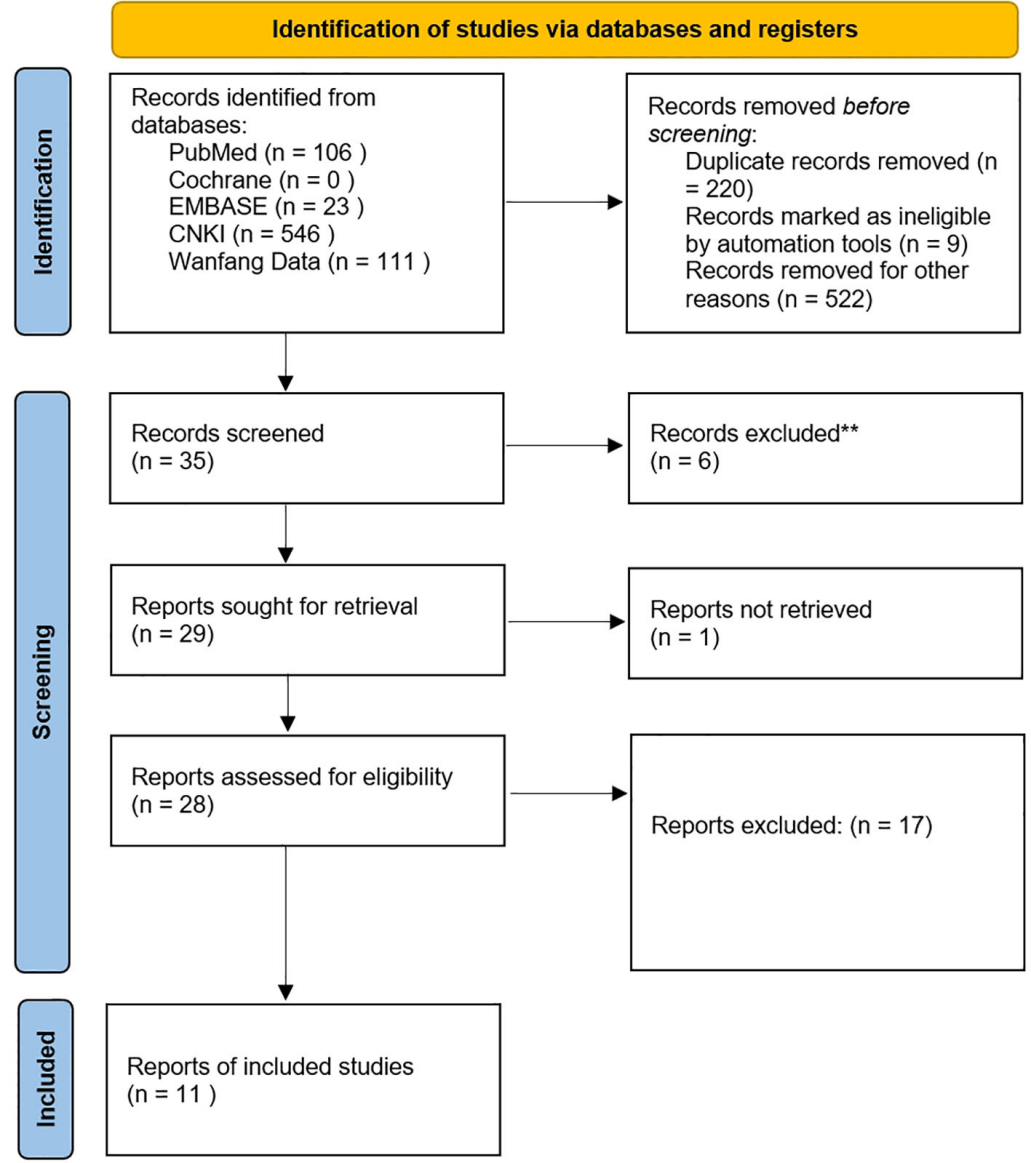
PRISMA Flow chart of article selection.

**Figure 2:**
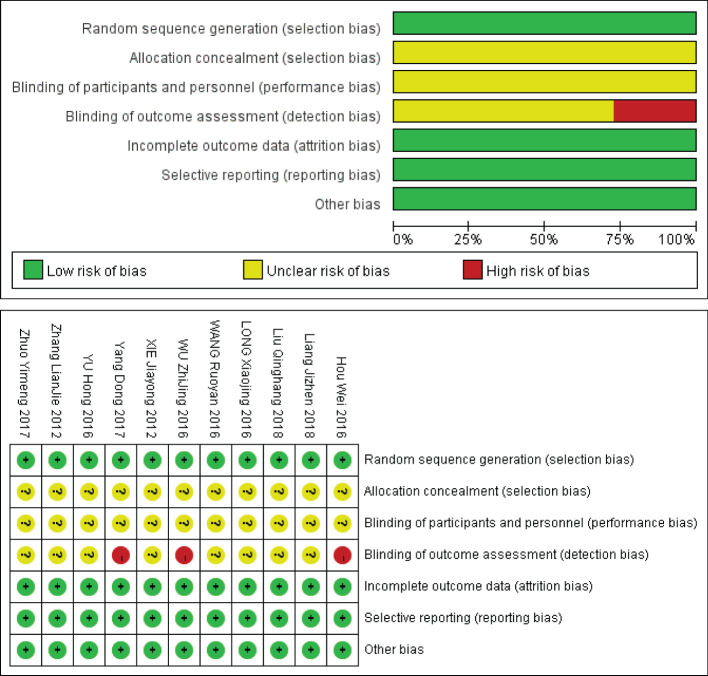
Evaluation results of methodology quality of included studies.

According to the retrieval strategy and manual retrieval, 786 literatures were initially obtained, and 11 literatures were finally included after screening layer by layer (9–19), with a total of 1216 patients, including 608 patients in MN group and 608 patients in PN group. The process and results of literature screening are shown in **Figure 1**. The basic characteristics of the study are shown in **Table 1**, and the results of bias risk assessment are shown in **Figure 2**.

**Table 1:** Basic characteristics of included studies (9–19).

Author	Year	Nutritional pathway	Sample	Age	Gender (M/F)	Outcome
Liu Qinghang (9)	2018	MN	60	61.71 ± 3.14	39/21	①②③④⑤
		PN	60	60.60 ± 3.14	37/23	
Zhuo Yimeng (10)	2017	MN	30	61.89 ± 9.36	20/10	②④⑥⑧⑨
		PN	30	62.37 ± 9.54	21/9	
WANG Ruoyan (11)	2016	MN	30	61.31 ± 5.88	19/11	①②③④⑧⑨
		PN	30	61.28 ± 5.84	21/9	
LONG Xiaojing (12)	2016	MN	40	56.70 ± 10.80	22/18	②③④⑤⑥⑦⑧⑨
		PN	40	54.20 ± 8.60	20/20	
Liang Jizhen (13)	2018	MN	20	65.75 ± 4.92	12/8	①②③
		PN	20	70.66 ± 4.74	8/12	
Hou Wei (14)	2016	MN	30	62.35 ± 6.36	35/25	②⑥⑦⑧
		PN	30			
Yang Dong (15)	2017	MN	30	66.40 ± 5.20	23/7	⑧⑨
		PN	30	65.60 ± 3.70	20/10	
WU ZhiJing (16)	2016	MN	45	68.76 ± 4.88	31/14	②⑥⑦⑧⑨
		PN	45	68.58 ± 4.92	30/15	
Zhang LianJie (17)	2012	MN	30	69.10 ± 3.22	23/7	②⑥⑧
		PN	30	68.59 ± 3.14	25/5	
YU Hong (18)	2016	MN	33	68.00 ± 33.30	16/17	①②④⑥⑦⑧⑨
		PN	33	66.20 ± 92.90	16/17	
XIE Jiayong (19)	2012	MN	290	54.70 ± 5.42	430/150	②③⑥⑨
		PN	290			

MN, Multi-nutrition; PN, Parenteral Nutrition; ①TRF, Transferrin; ②ALB, Albumin; ③PA, prealbumin; ④LYM, absolute lymphocyte count; ⑤IgA/G/M, immunoglobulin A/G/M; ⑥Anal exhaust recovery time; ⑦Defecation time; ⑧Hospitalized time; ⑨Adverse events (lung infection, abdominal distension, incision infection, anastomotic leakage).

### Meta-analysis results

Postoperative laboratory tests included Transferrin level Albumin level, Prealbumin level, Absolute lymphocyte count level and IgA, IgG, IgM levels. Clinical symptom remission includes Anal exhaust recovery time, Defecation time, and Hospitalization time. Adverse event included pneumonia, abdominal distension, wound infection, and anastomotic leakage. The result looks like this:

### Postoperative transferrin level

**Figure 3:**
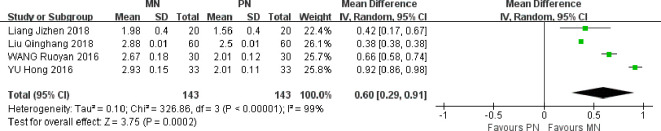
Meta-analysis of comparison of postoperative TRF levels.

Four studies were included. Meta-analysis of randomized effect model showed that compared with PN group, the transferrin level of esophageal cancer patients in MN group was higher on the 7th day after operation, and the difference between the two groups was statistically significant [MD = 0.60, 95% CI (0.29, 0.91), *P* = 0.0002]. The serum transferrin level of MN group was higher than that of the control group, which indicated that enteral and parenteral nutrition support could improve the nutritional status of patients, further improve the immune function, provide support for the heart and other organs, and reduce the occurrence of related complications, as shown in **Figure 3**.

### Postoperative albumin level

**Figure 4:**
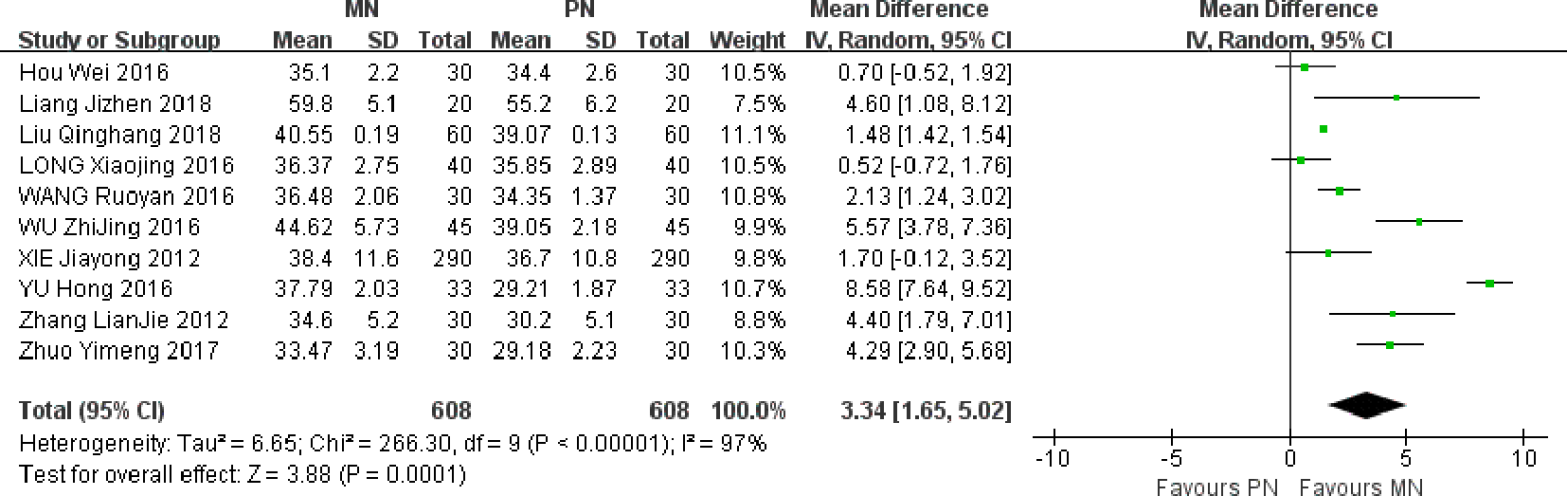
Meta-analysis of comparison of postoperative ALB levels.

A total of 10 studies were included. Meta-analysis of randomized effect model showed that the improvement degree of serum albumin in MN group was better than that in PN group on the 7th day after operation, and the difference between the two groups was statistically significant [MD = 3.34, 95% CI (1.65, 5.02), *P* = 0.0001]. The serum albumin level of MN group was higher than that of the control group, indicating that enteral and parenteral nutrition support can improve the nutritional status of patients and further improve immune function, as shown in **Figure 4**.

### Postoperative prealbumin level

**Figure 5:**
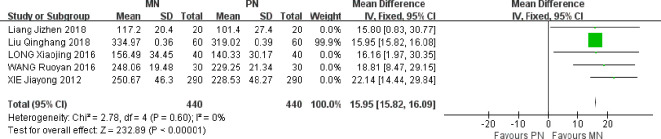
Meta-analysis of comparison of postoperative PA levels.

Five studies were included. Meta-analysis of fixed effect model showed that compared with PN group, the serum prealbumin level of esophageal cancer patients in MN group was higher on the 7th day after operation, and the difference between the two groups was statistically significant [MD = 15.95, 95% CI (15.2, 16.09), *P* < 0.00001]. Like albumin, the nutritional status of patients with prealbumin reaction in the past two days and the level of prealbumin after surgery increased, indicating that the MN group can improve the nutritional status of patients and further improve immune function, as shown in **Figure 5**.

### Postoperative absolute lymphocyte count level

**Figure 6:**
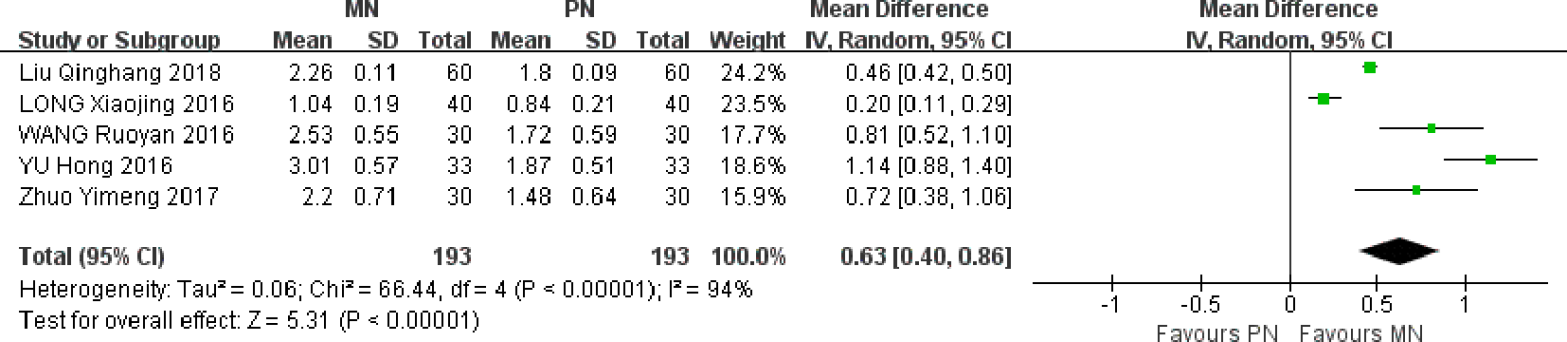
Meta-analysis of comparison of postoperative LYM levels.

Five studies were included. Meta-analysis of random effect model showed that the improvement of lymphocyte count in MN group was better than that in PN group on the 7th day after operation, and the difference between the two groups was statistically significant [MD = 0.63, 95% CI (0.40, 0.86), *P* < 0.00001]. Postoperative lymphocyte count level also reflects the nutritional status of patients, MN group lymphocyte count level is higher than the control group, indicating that enteral and parenteral nutrition support can improve the nutritional status of patients, and further improve immune function, as shown in **Figure 6**.

### Postoperative IgA, IgG, IgM level

IgA results were included in 2 studies. Meta-analysis of fixed effect model showed that the improvement degree of IgA in MN group was better than that in PN group on the 7th day after operation, and the difference between the two groups was statistically significant [MD = 0.38, 95% CI (0.35, 0.41), *P* < 0.00001], as shown in **Table 2**.

**Table 2:** Meta-analysis of comparison of postoperative IgA、IgG、IgM levels.

Outcomes	Studies	Heterogeneity test results		Effect model	Meta-analysis results	
		*P*	*I^2^*		95%CI	*P*
IgA	2	0.18	44%	Fixed	0.38 [0.35, 0.41]	< 0.00001
IgG	2	0.36	0%	Fixed	1.80 [1.60, 2.00]	< 0.00001
IgM	2	0.12	59%	Random	0.39 [0.15, 0.62]	0.001

IgG results were included in 2 studies. Meta-analysis of fixed effect model showed that the improvement degree of IgG in MN group was better than that in PN group on the 7th day after operation, and the difference between the two groups was statistically significant [MD = 1.80, 95% CI (1.60, 2.00), *P* < 0.00001], as shown in **Table 2**.

IgM results were included in 2 studies. Meta-analysis of randomized effect model showed that the improvement degree of IgM in MN group was better than that in PN group on the 7th day after operation, and the difference between the two groups was statistically significant [MD = 0.39, 95% CI (0.15, 0.62), *P* = 0.001], as shown in **Table 2**.

IgA, IgG, IgM are important antibodies for human humoral immunity. This result shows that early enteral nutrition combined with parenteral nutrition is beneficial to the recovery of patients’ immune function and reduce immune harm.

### Anal exhaust recovery time

**Figure 7:**
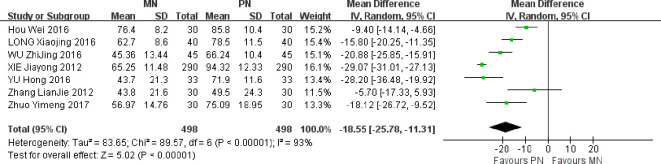
Meta-analysis of comparison of postoperative Anal exhaust recovery time.

A total of 7 studies were included. Meta-analysis of random effect model showed that compared with PN group, the anal exhaust recovery time of esophageal cancer patients in MN group was shorter after operation, and the difference between the two groups was statistically significant [MD = -18.55, 95% CI (-25.78, -11.31), *P* < 0.00001]. The anal exhaust time in MN group was significantly shortened, which showed that the MN group program was conducive to improving the gastrointestinal function of patients, as shown in **Figure 7**.

### Defecation time

**Figure 8:**
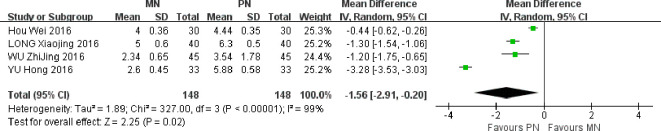
Meta-analysis of comparison of postoperative Defecation time.

Four studies were included. Meta-analysis of randomized effect model showed that compared with PN group, the postoperative defecation time of esophageal cancer patients in MN group was shorter, and the difference between the two groups was statistically significant [MD = -1. 56, 95% CI (-2.91, -0.20), *P* = 0.02]. The defecation time of MN group patients was significantly shortened, indicating that the MN group regimen is beneficial for improving the gastrointestinal function of patients, as shown in **Figure 8**.

### Hospitalization time

**Figure 9:**
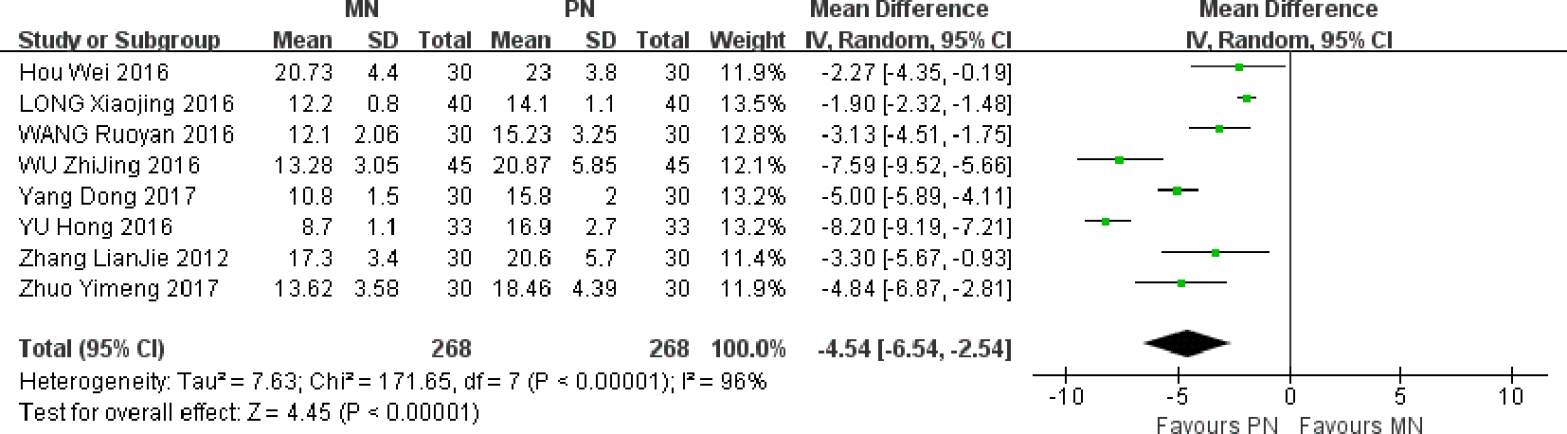
Meta-analysis of comparison of postoperative Hospitalization time.

A total of 8 studies were included. Meta-analysis of randomized effect model showed that compared with PN group, the postoperative hospitalization time of esophageal cancer patients in MN group was shorter, and the difference between the two groups was statistically significant [MD = -4. 54, 95% CI (-6.54,-2.54), *P* < 0.00001]. The hospitalization time of patients in MN group was significantly shortened, which shows that the scheme of MN group is conducive to improving the gastrointestinal function of patients, as shown in **Figure 9**.

### Adverse event

Adverse events included pneumonia, abdominal distension, wound infection and anastomotic leakage. Meta-analysis showed that the risk of postoperative pneumonia [RR = 0.32, 95% CI (0.20, 0.53, *P* < 0.00001), abdominal distension [RR = 0.53, 95% CI (0.24, 1.16), *P* = 0.11], wound infection [RR = 0.37, 95% CI (0.16, 0.87), *P* = 0. 02], anastomotic fistula [RR = 0. 33, 95% CI (0.12, 0.96), *P* = 0.04] in MN group were lower than those in PN group. The results of pneumonia, wound infection and anastomotic fistula were statistically significant, as shown in **Table 3**.

**Table 3:** Meta-analysis of comparison of postoperative Adverse event.

Outcomes	Studies	Heterogeneity test results		Effect model	Meta-analysis results	
		*P*	*I^2^*		95%CI	*P*
Pneumonia	7	0.84	0%	Fixed	0.32 [0.20, 0.53]	< 0.00001
Abdominal Distention	3	0.88	0%	Fixed	0.53 [0.24, 1.16]	0.11
Incision infection	3	0.96	0%	Fixed	0.37 [0.16, 0.87]	0.02
Anastomotic fistula	5	0.80	0%	Fixed	0.33 [0.12, 0.96]	0.04

### Publication bias test

**Figure 10:**
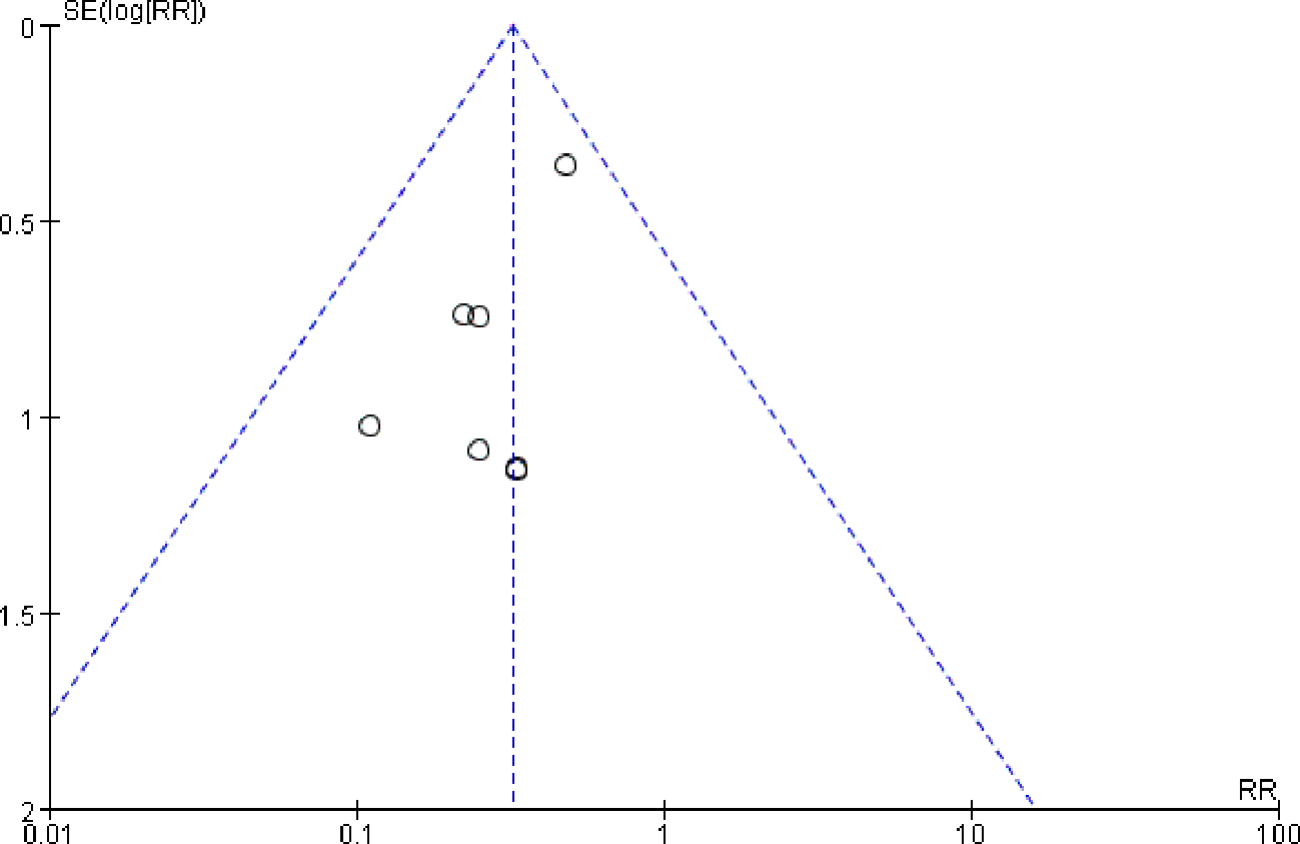
Funnel analysis of the incidence of pulmonary infection.

The impact of publication bias on Meta-analysis results was analyzed by Funnel plot. Taking the incidence of pulmonary infection as an example, the results showed that the included studies were basically evenly distributed on both sides of funnel diagram, suggesting that publication bias had little impact on the research results in this study, as shown in **Figure 10**.

## Discussion

PN and Enteral nutrition (EN) are commonly used in nutritional support therapy. PN is widely used in clinical practice, which can effectively improve the nutritional status of the body. However, PN does not conform to the physiological state. Long-term application is easy to cause intestinal mucosal atrophy, intestinal morphology and function disorder, and immune function decline (20). The perioperative recovery of patients is greatly affected. Because of the decline of immune function, it may cause tumor recurrence and metastasis, intestinal flora shift, and even intestinal septicemia. EN has the advantages of conforming to physiological state, maintaining intestinal needs and functional integrity, etc., and is widely used in clinical practice.

Good nutritional status is an important guarantee to ensure the smooth recovery of patients after operation. Esophageal cancer has difficulty swallowing before operation, and there are different degrees of malnutrition in tumor consumption. After operation, due to large surgical trauma, it is in a state of high decomposition, and malnutrition is obviously aggravated, which is easy to induce complications such as infection and anastomotic leakage (21). Therefore, postoperative nutritional support for esophageal cancer is more important than any other postoperative nutritional support. Postoperative gastrointestinal paralysis is limited to stomach and colon, and the peristalsis and absorption function of small intestine have recovered early after operation. At the same time, the peristalsis, digestion and absorption functions of small intestine can be restored within a few hours after operation. The absorption of large nutrients can be completed in the small intestine, which provides a theoretical basis for early enteral nutrition after esophageal cancer surgery. Slow dripping of normal saline at 6 hours after operation can also promote the recovery of intestinal peristalsis. Therefore, early enteral nutrition is particularly important. Compared with PN, early enteral nutrition has the following advantages: the absorption of nutrients in early enteral nutrition through portal vein system is an active process, which is conducive to protein synthesis and metabolic regulation of internal organs (especially liver), conforms to physiological conditions, can self-regulate, is conducive to maintaining the integrity of intestinal mucosa, maintaining intestinal mucosal barrier, reducing surgical stress, improving patients’ nutritional status and immune function, shortening postoperative recovery time, increasing nutritional intake, reducing albumin decomposition and improving nutritional status (22). It plays an important role in postoperative physical recovery, prevention of infection, anastomotic leakage and promotion of rehabilitation (23). However, during the implementation of early enteral nutrition, patients have feeding intolerance such as increased gastric residue, abdominal distension, diarrhea and vomiting. Mentec H et al. (24) showed that the incidence of intolerance is about 46%. Early enteral nutrition intolerance will not only reduce patients’ comfort and prolong patients’ energy reaching the standard, but also increase patients’ risk of infection by switching to or relying solely on PN for nutritional support. At this time, PN is used in combination, that is, from EEN + PN = MN, and then transits to total enteral nutrition, emphasizing the combination of EN and PN in the early stage, and gradually transits to full EN and then stops PN. Providing insufficient energy and protein through PN has been clinically recognized, reaching the target requirement of the body, which is beneficial to the normal metabolism of human tissues and maintaining organ function (25).

From the results of this Meta-analysis, compared with PN group, MN group can improve the levels of transferrin, serum albumin and serum prealbumin after operation. On the one hand, enteral nutrition in MN group can make nutrients directly reach and absorb through the small intestine, stimulate the growth of small intestinal mucosal cells and maintain the integrity of its structure and function. In addition, enteral nutrition can increase the blood flow of portal vein, provide more abundant raw materials for liver protein synthesis, and promote liver albumin and prealbumin synthesis, which is more in line with the physiological mode of nutrition absorption compared with parenteral nutrition. On the other hand, PN provides insufficient energy and protein to achieve the target demand of the body, promote normal metabolism and maintain organ function. In gastrointestinal function, compared with PN group, MN group can shorten the anal recovery time, defecation time and hospitalization time after operation. In MN group, early enteral nutrition can shorten the recovery time of intestinal function, and enteral nutrition support can increase intestinal motor function, intestinal blood flow and liver blood perfusion, effectively synthesize protein and short chain fatty acids, and improve liver function of patients. The levels of LYM, IgA, IgG, IgM in MN group were higher than those in PN group (P < 0.05), indicating that enteral nutrition combined with parenteral nutrition can effectively improve the immune function of patients.

In terms of postoperative adverse events, compared with PN group, MN group can reduce the incidence of lung infection, incision infection, abdominal distension and anastomotic leakage, which is mainly because mixed nutritional support can effectively improve the nutritional status of patients and promote the recovery of gastrointestinal function, thus promoting the recovery of the body and reducing complications; In addition, the infusion volume of MN group was significantly less than that of PN group, which was also a factor to reduce lung infection.

This study also has some limitations. First, the sample size included in the original literature is small and the data are limited. The bias caused by small sample size is mainly reflected in reducing the reliability of conclusions, affecting statistical effectiveness, and increasing errors. When the sample size is insufficient, the reliability of the research conclusions will be reduced. It may also cause the research results to deviate from the real situation, making the reliability of the conclusions questioned. At the same time, insufficient sample size can also affect statistical potency, that is, the ability of a study to correctly detect actual effects. In the case of small sample size, the influence of random error and bias may be more significant, making the study results deviate from the real situation. The small sample size included in this study is also one of the important reasons for the high heterogeneity, and it is also one of the potential sources of bias. Secondly, many important details of the included studies, such as involving heterogeneous populations, limit our further analysis to a certain extent. More studies still need to be included in subsequent studies to improve. However, it is worth noting that the feasibility and scientificity of the ideas of this study and the objectivity and reliability of the results of this study indicate that early postoperative support treatment with enteral nutrition and parenteral nutrition for patients with esophageal cancer can Better guarantee the nutritional intake of patients, improve the nutritional status of patients, and promote the recovery of patients after surgery. In addition, the results and limitations of this study will also provide more detailed suggestions for future research.

## Conclusion

This Meta-analysis is an observational study, and there must be biases in the process of design, data collection, and statistical analysis, such as publication bias and language bias. Therefore, the limitations of Meta-analysis method may have a certain impact on the reliability of comprehensive analysis results. The current evidence in this paper shows that mixed feeding can improve the nutritional status of patients after surgery, improve the immune function of patients, shorten the anal exhaust time, defecation time, postoperative hospital stay, and reduce the incidence of postoperative complications. Limited by the quality of the included literature research, the above conclusions need to be further verified by large-sample and high-quality RCT.

## Data availability statement

The original contributions presented in the study are included in the article/supplementary material. Further inquiries can be directed to the corresponding authors.

## Author contributions

SL: Writing – original draft, Writing – review & editing. LQ: Data curation, Writing – review & editing. YaL: Data curation, Writing – review & editing. HL: Data curation, Writing – review & editing. YiL: Writing – review & editing. JS: Writing – review & editing. WC: Resources, Writing – review & editing. RS: Resources, Writing – review & editing. LZ: Data curation, Writing – review & editing. XL: Funding acquisition, Writing – review & editing.
